# Leucine-enriched essential amino acids attenuate muscle soreness and improve muscle protein synthesis after eccentric contractions in rats

**DOI:** 10.1007/s00726-015-1946-9

**Published:** 2015-03-14

**Authors:** Hiroyuki Kato, Hiromi Suzuki, Masako Mimura, Yoshiko Inoue, Mayu Sugita, Katsuya Suzuki, Hisamine Kobayashi

**Affiliations:** Institute for Innovation, Ajinomoto Co., Inc., Kawasaki, Kanagawa 210-8681 Japan

**Keywords:** Eccentric contractions, Muscle protein synthesis, Muscle soreness, Leucine-enriched essential amino acids, mTOR

## Abstract

Eccentric exercise results in prolonged muscle weakness and muscle soreness, which are typical symptoms of muscle damage. Recovery from muscle damage is related to mammalian target of rapamycin (mTOR) activity. Leucine-enriched essential amino acids (LEAAs) stimulate muscle protein synthesis via activation of the mTOR pathway. Therefore, we investigated the effect of LEAAs on muscle protein synthesis and muscle soreness after eccentric contractions (EC). Male Sprague–Dawley rats (9–11 weeks old) were administered an LEAA solution (AminoL40; containing 40 % leucine and 60 % other essential amino acids) at 1 g/kg body weight or distilled water (control) 30 min before and 10 min after EC. Tibialis anterior (TA) muscle was exposed to 500 EC by electrical stimulation under anesthesia. The fractional synthesis rate (FSR; %/h) in the TA muscle was measured by incorporating l-[ring-^2^H_5_] phenylalanine into skeletal muscle protein. Muscle soreness was evaluated by the paw withdrawal threshold using the Randal–Selitto test with some modifications from 1 to 3 days after EC. The FSR in the EC-control group (0.147 ± 0.016 %/h) was significantly lower than in the sedentary group (0.188 ± 0.016 %/h, *p* < 0.05). AminoL40 administration significantly mitigated the EC-induced impairment of the FSR (0.172 ± 0.018 %/h). EC decreased the paw withdrawal threshold at 1 and 2 days after EC, which indicated that EC induced muscle soreness. Furthermore, AminoL40 administration alleviated the decreased paw withdrawal threshold. These findings suggest that LEAA supplementation improves the rate of muscle protein synthesis and ameliorates muscle soreness after eccentric exercise.

## Introduction

The importance of muscle mass, strength, and metabolic function in athletic performance, daily activities, and general health is widely recognized. Resistance exercise increases muscle size and strength. The effect of resistance exercise on skeletal muscle growth depends on the mode of contraction and duration, intensity, and frequency of exercise (Tesch 1988). Several studies have compared the effects of resistance exercise with concentric and/or eccentric contractions (ECs), showing that eccentric exercise results in greater gains in muscle strength and size than concentric exercise because of greater overload induced by ECs (Higbie et al. 1996). Therefore, eccentric exercise is considered important for exercise-induced muscle hypertrophy.

However, eccentric exercise-biased training is not widely used, partly because eccentric exercise has some adverse effects. Eccentric exercise results in prolonged muscle weakness and muscle soreness (Proske and Morgan 2001), which are symptoms of muscle damage. This muscle soreness gradually develops and lasts for several days (Nosaka 2007). Muscle soreness and muscle weakness reduce the ability to perform athletic activities and potentially prevent regular exercise (Cleak and Eston 1992). Therefore, to make eccentric exercise possible for various practical uses, (e.g., promoting general health and athletic performance), an effective intervention that decreases the adverse effects, and augments the positive effects of the eccentric exercise, needs to be identified.

The initial phase of the recovery process from muscle damage is characterized by inflammation and degeneration of damaged tissue. Satellite cells are then activated, and they proliferate, differentiate, and fuse to myofibrils to repair muscle tissue (Charge and Rudnicki 2004). The recovery process is regulated by intracellular signaling pathways that control protein turnover, maintaining a balance between muscle protein synthesis and muscle protein degradation. Among these pathways, the mammalian target of rapamycin (mTOR) pathway is an essential step for muscle regeneration (Ge et al. 2009).

Amino acids are known to be an important nutrient for stimulation of muscle protein synthesis. Resistance exercise and feeding of amino acids have a marked stimulatory effect on the rate of muscle protein synthesis (Biolo et al. 1997). Among amino acids, essential amino acids play an important role in increasing the rate of muscle protein synthesis (Volpi et al. 2003). Among essential amino acids, leucine in particular plays a specific role in stimulation of the mTOR pathway (Crozier et al. 2005). However, administration of leucine alone or a branched chain amino acids (BCAAs) mixture leads to a decrease in plasma concentrations of other essential amino acids in neonatal pigs (Escobar et al. 2005) and in humans (Borgenvik et al. 2012). In normal swine, reduced availability of amino acids leads to reduced muscle protein synthesis and amino acid supplementation reverses this effect (Kobayashi et al. 2003). Furthermore, insulin-mediated hypoaminoacidemia reduces protein synthesis; this is mitigated by increased availability of all amino acids, but not leucine alone. Therefore, essential amino acids, other than BCAAs and leucine may be essential for sustaining muscle protein synthesis. Accordingly, leucine-enriched essential amino acids (LEAAs) increase the rate of muscle protein synthesis after several types of exercise (Dreyer et al. 2008; Pasiakos et al. 2011). However, there are no reports on the effect of LEAAs on recovery from muscle soreness after eccentric exercise. Some reports have shown that administration of amino acids (Nosaka et al. 2006), particularly leucine (Kirby et al. 2012) or BCAAs (Jackman et al. 2010), administration suppresses delayed-onset muscle soreness (DOMS) 1 day after exercise in humans. However, the mechanism by which amino acids act to decrease the degree of muscle soreness, and the optimal composition of amino acids to reduce the muscle soreness, remain unclear.

Wide individual variability of muscle soreness after eccentric exercise have been reported (Gulbin and Gaffney 2002), and this creates a problem in assessing the effects of prophylactic or therapeutic interventions on muscle soreness. The wide individual variability is not attributed to genetic variability, and may be attributed to the preconditioning induced by daily muscle contractile activity (Gulbin and Gaffney 2002). Thus, in order to investigate the optimal composition of amino acid to decrease muscle soreness or the mechanism by which amino acids acts to decrease muscle soreness, the animal model in which muscle contractile activity can be properly controlled, is required.

The purpose of this study was to investigate: (1) the effect of LEAAs on muscle soreness, which is a typical symptom of exercise-induced muscle damage, and (2) the effect of LEAAs on the rate of muscle protein synthesis after ECs in rats. The tibialis anterior (TA) muscle of rats was forced to contract by electrical stimulation. The fractional synthesis rate (FSR; %/h) was determined by calculating the incorporation rate of L-[ring-^2^H_5_] phenylalanine into the skeletal mixed muscle protein pool. Muscle soreness was evaluated by measuring the mechanical paw withdrawal threshold according to the pressure stimulus.

## Materials and methods

### Animals

This study was approved by the Institutional Animal Care and Use Committee of Ajinomoto Co., Inc. Male Sprague–Dawley rats (300–350 g; Charles River Laboratories Japan, Inc., Yokohama, Japan) were used in this study. The rats were housed in a temperature-controlled room on a 12-h light–dark cycle. They were also provided a standard commercial chow (CR-F1; Charles River Laboratories Japan, Inc., Yokohama, Japan) and water was provided ad libitum throughout the experiment.

### Experimental design

*Experiment 1* The effect of LEAAs on the FSR in the TA muscle after ECs was investigated in rats after 3 h of food deprivation. The rats were divided into one of three groups: Sed, sedentary controls and administration of distilled water as a control (*n* = 9); EC-Con, ECs and administration of distilled water as a control (*n* = 10); EC-AminoL40, ECs and administration of LEAAs (*n* = 9). The TA muscle was stimulated electrically via needle electrodes that were inserted near the common peroneal nerve under anesthesia with sodium pentobarbital (50 mg/kg, i.p.). Electrical stimulation was applied for 1 s with current strength, which is three times as much as the twitch threshold (<100 μA), and a frequency of 50 Hz with a pulse duration of 1 ms was used (Taguchi et al. 2005). The TA muscle was simultaneously stretched with electrical stimulation from an ankle position of 45° to 135° over a 1-s period with the use of a linearized servomotor (CPL28T2B-06KD, OrientalMotor Co. Ltd, Tokyo, Japan), and then returned to the starting position over 3 s (ankle position was defined as the angle between the tibia and the plantar surface of the foot, with 180° representing a completely extended foot). The electrical stimulation was repeated every 4 s for a total of 500 repetitions. EC-AminoL40 group was administered LEAAs (1 g/kg body weight) by oral gavage 30 min before and 10 min after EC because of following. (1) It has been reported that some types of amino acid supplementation (e.g., leucine alone, BCAAs or a 12 amino acids mixture) before and immediately after exercise may attenuate muscle soreness (Jackman et al. 2010; Kirby et al. 2012; Nosaka et al. 2006). (2) It has been reported that plasma amino acid concentrations reached a level sufficient to stimulate the mTOR pathway 30 min after ingestion of a 0.3375 or 0.675 g/kg leucine (Crozier et al. 2005). (3) After EC, we administered the amino acids as soon as the rats awoke from anesthesia, which took 10 min. The AminoL40 mixture consisted of essential amino acids in the following proportions: histidine, 2 %; isoleucine, 11 %; leucine, 40 %; lysine, 17 %; methionine, 3 %; phenylalanine, 7 %; threonine, 9 %; tryptophan, 1 %; and valine, 11 % (Except for the higher proportion of leucine, this mixture contains the ratio of essential amino acids found in whey protein; all amino acids were manufactured by Ajinomoto Co., Inc., Tokyo, Japan). The AminoL40 mixture was developed with the specific purpose of avoiding substantially decreasing the availability of the other EAAs while increasing the proportion of leucine. Both the Sed and EC-Con groups were administrated distilled water as a control. The FSR was evaluated using the flooding dose method as described by Garlick and McNurlan (1998). Briefly, 30 min after second oral administration, rats in all groups were injected with flooding doses of phenylalanine (1.5 mmol/kg body weight) containing l-[ring-^2^H_5_]-phenylalanine (50 MPE, Cambridge isotope, Cambridge, MA) iv into the tail vein. 20 min after the phenylalanine injection, blood was collected from the abdominal aorta, the TA muscle was then removed under anesthesia, and frozen in liquid nitrogen and stored at −80 °C. Blood was separated from plasma by centrifugation at 10,000×*g* for 10 min at 4 °C, and the plasma was stored at −80 °C.

*Experiment 2* EC and administration of LEAAs or distilled water were applied under the same conditions as experiment 1 (EC-Con and EC-AminoL40 groups, *n* = 11 per group). Muscle soreness was evaluated by mechanical paw withdrawal threshold as previously described (Taguchi et al. 2005). Briefly, a Randall–Selitto analgesiometer (Ugo Basile, Italy) equipped with a probe with a tip diameter of 2.6 mm was used to measure mechanical paw withdrawal threshold. The TA muscle was pushed by the probe through shaved skin. The speed of force increment was set at 157 mN/s. The intensity of pressure causing an escape reaction was defined as the mechanical paw withdrawal threshold. The mechanical paw withdrawal threshold was evaluated before, and 1, 2, and 3 days after EC.

### Measurement of the FSR

Approximately 30 mg of TA muscle was homogenized in 15 % sulfosalicylic acid, and the homogenate was centrifuged at 10,000×*g* for 10 min at 4 °C. The supernatant was used for measurement of enrichment of free phenylalanine in TA muscle. The precipitate, which was hydrolyzed in 2 ml of 6 N hydrochloric acid at 90 °C for 16 h, was used for measurement of enrichment of protein-bound phenylalanine in TA muscle. Amino acids in the supernatant and the hydrolysate were purified using cation exchange chromatography (Dowex 50 W 8X; Bio-Rad Laboratories, Hercules, CA), and dried in a rotary evaporator (Nakajima Corp., Tokyo Japan). Phenylalanine enrichment (*E*_(muscle free)_) in the supernatant was determined by its *tert*-butyl dimethylsilyl derivatization (Thermo Fisher Scientific, Waltham, MA). Gas chromatography–mass spectrometry was used to monitor ions 336 and 341 in the electron impact mode (GC–MS; 6890 GC system and 5473 Network Mass Selective Detector, Agilent, Santa Clara, CA). Muscle protein-bound phenylalanine enrichment (*E*_(protein-bound)_) was determined by measuring the butyl derivatization (HCl-*n*-butanol [10 v/v %]: GL Science Inc., Japan) using liquid chromatography–mass spectrometry to monitor ions 224 and 227 in the first mass spectrometry, and 122 and 125 in the second mass spectrometry (LC–MS/MS; Prominence HPLC system, Shimazu, Kyoto, Japan and API 3200, Applied Biosystems, Carlsbad, CA) using the external standard curve approach (Calder et al. 1992).

### Immunoblot analysis

Muscles were homogenized on ice in a five times volume of homogenization buffer [25 mM Tris–HCl (pH 7.6), 1 % NP-40, 0.5 % sodium deoxycholate, 0.1 % sodium dodecyl sulfate (SDS), 150 mM NaCl, 1 % protease inhibitor cocktail and 1 % phosphatase inhibitor (Sigma Aldrich, St. Louis, MO)]. The homogenates were separated by centrifugation at 19,800×*g* for 30 min at 4 °C. Protein concentrations were assessed in duplicate using the BCA protein assay kit (Pierce Biotechnology, Rockford, IL). Muscle homogenates were solubilized in sample loading buffer (50 mM Tris–HCl [pH 6.8], 10 % glycerol, 6 % beta-mercaptoethanol, 2 % SDS, and 0.1 % bromophenol blue) at a concentration of 7.5 mg/ml and boiled for 3 min. Samples were loaded on SDS–polyacrylamide gels [TGX 4–15 % gradient gel (Bio-Rad, Hercules, CA)]. Protein was then separated by electrophoresis (200 V for 40 min at room temperature).

Separated proteins were transferred onto a polyvinylidene fluoride membrane (no. 162-0176; Bio-Rad, Hercules, CA) at 200 mA in transfer buffer (25 mM Tris-base, 192 mM glycine, and 20 % methanol). The membranes were blocked for 1 h at room temperature in TBS-T (20 mM Tris-base, 150 mM NaCl, and 0.1 % Tween-20) containing 5 % bovine serum albumin (BSA), and were serially washed in TBS-T at room temperature. Membranes were then probed for specific signaling proteins using antibodies for detection of p70S6K1 and phospho-p70S6K1 (Thr 389). All antibodies were purchased from Cell Signaling Technology (Beverly, MA). Membranes were incubated overnight at 4 °C in primary antibody buffer (5 % BSA in TBS-T, pH 7.6, primary antibody diluted 1:1000). The membranes were serially washed in TBS-T, incubated with horseradish peroxidase-conjugated secondary antibody (dilution, 1:10,000) in TBS-T for 1 h, and serially washed in TBS-T. Horseradish peroxidase activity was detected using enhanced chemiluminescence reagent (Prime Western Blotting Detection System; Bio-Rad, Hercules, CA). Optical density measurements were obtained with densitometric scanning using the LAS3000 (Fuji Film, Tokyo, Japan). Membranes containing phospho-detected proteins were stripped of primary and secondary antibodies by use of stripping buffer (Nacalai Tesque, Kyoto, Japan). 1 h after incubation in stripping buffer, membranes were reprobed for total protein with the specific antibody of interest. Immunoblot data are expressed as normalized phosphor-protein divided by total protein (phospho/total).

### Measurements of blood variables

Insulin is a powerful stimulator of protein synthesis, and leucine is known to stimulate insulin secretion (Crozier et al. 2005). Therefore, to investigate the relationship between the stimulation of the insulin secretion by AminoL40 and skeletal muscle protein synthesis, plasma insulin concentrations were measured using a commercial ELISA kit (Morinaga Institute Biological Science, Yokohama, Japan). Plasma amino acid concentrations were measured with an automatic amino acid analyzer (JLC-500; JEOL, Tokyo, Japan).

### Calculations

The FSR of TA muscle protein was calculated with the precursor–product model. The enrichment of precursor was represented by* E*_(muscle free)_, and the enrichment of product was represented by* E*_(protein-bound)_. Muscle protein synthesis was calculated as FSR (%/h) = *E*_(protein-bound)_/(*E*_(muscle free)_ × *t*) × 100, where *t* represents the time interval between phenylalanine injection and tissue sampling.

### Statistical analysis

Values are shown as mean ± SE. A repeated measures two-way ANOVA followed by Bonferroni’s multiple-comparisons test was performed to test the mechanical withdrawal threshold with LEAA administration and various time points were the independent variables (GraphPad Prism; GraphPad Software Inc., San Diego, CA). Changes in the other measurements were examined with one-way ANOVA followed by Tukey’s multiple-comparisons test. Values of* p* < 0.05 were considered significant.

## Results

### Measurement of the FSR

**Fig. 1:**
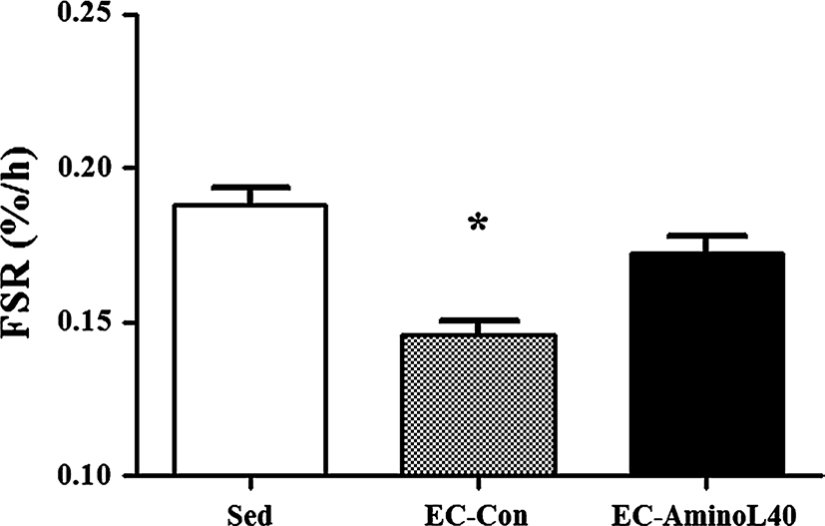
Effect of EC and administration of AminoL40 on the FSR (%/h) of TA muscle protein. The FSR in TA muscle protein was significantly lower in the EC-Con group compared with the Sed group. The FSR was significantly higher in the EC-AminoL40 group compared with the EC-Con group. There was no significant difference in the FSR between the Sed and EC-AminoL40 groups. Data are shown as mean ± SE (*n* = 9 for the Sed and EC-AminoL40 groups, and *n* = 10 for the EC-Con group). *p < 0.05 significantly different from other groups

The FSR in TA muscle protein was significantly lower in the EC-Con group compared with the Sed group (Fig. 1, *p* < 0.05). The FSR was significantly higher in the EC-AminoL40 group compared with the EC-Con group (Fig. 1, *p* < 0.05). There was no significant difference in the FSR between the Sed and EC-AminoL40 groups.

### Immunoblot analysis

**Fig. 2:**
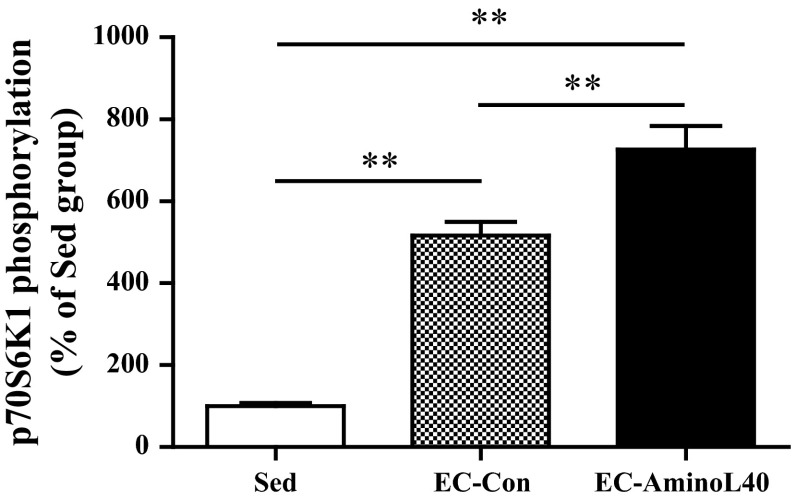
Effect of EC and administration of AminoL40 on the relative phosphorylation state of ribosomal S6 protein kinase 1 (phospho-S6K1 [Thr389]/total S6K1) in TA muscle. Phosphorylation of p70S6K1 was higher in the EC-Con group than the Sed group. Administration of AminoL40 further increased phosphorylation. Values are arbitrary units and presented as mean ± SE (*n* = 9 for the Sed and EC-AminoL40 groups, and *n* = 10 for the EC-Con group). **p < 0.01 between groups

The phosphorylation of p70S6K1 at Thr389 was significantly higher in the EC-Con group compared with the Sed group (Fig. 2, *p* < 0.01). There was a further increase in phosphorylation of p70S6K1 at Thr389 in the EC-AminoL40 group compared with the EC-Con group (Fig. 2, *p* < 0.01).

### Blood variables

**Fig. 3:**
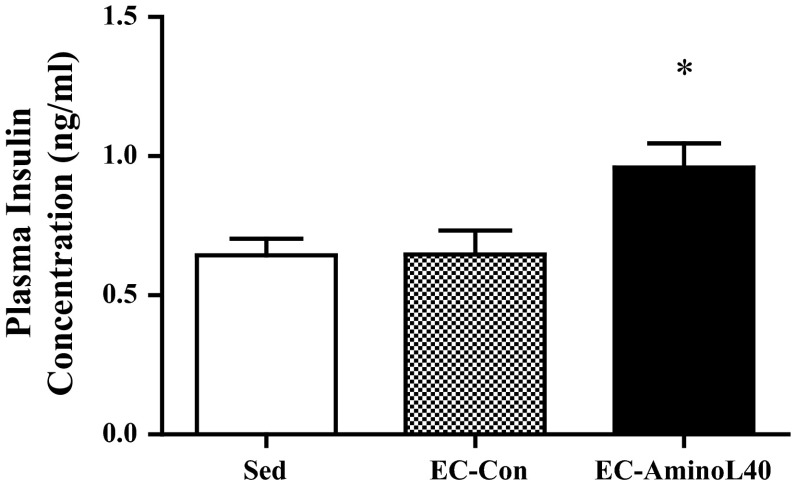
Effect of EC and administration of AminoL40 on plasma insulin concentrations. Insulin concentrations did not differ between the Sed and EC-Con groups, but were significantly higher in the EC-AminoL40 group compared with both the Sed and EC-Con groups. Data are shown as mean ± SE (*n* = 9 for the Sed and EC-AminoL40 groups, and *n* = 10 for the EC-Con group) *p < 0.05 significantly different from other groups

**Fig. 4:**
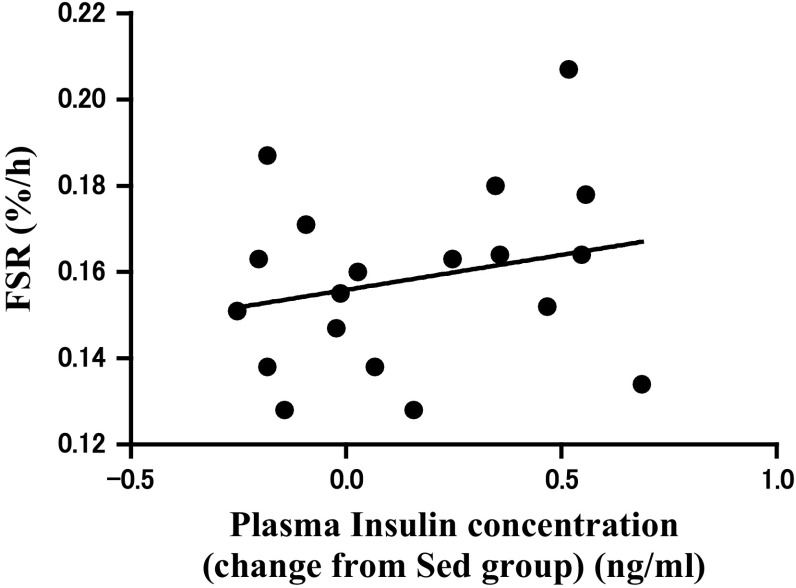
Correlation between the FSR in TA muscle and change in plasma insulin concentrations from Sed group. The 19 data points from EC-Con and EC-AminoL40 have been pooled in this figure. There was no significant correlation between the FSR in TA muscle and change in plasma insulin concentrations (*R* = 0.24, *p* = 0.33)

Plasma insulin levels were significantly higher in the EC-AminoL40 group than in the EC-Con and Sed groups (Fig. 3, *p* < 0.05). Furthermore, the data points from EC-Con and EC-AminoL40 groups have been pooled in Fig. 4; the data of EC-Con and EC-AminoL40 groups were pooled to examine the relationship between plasma insulin and FSR. There was no correlation between changes in plasma insulin concentrations from Sed group and FSR (*R* = 0.24, *p* = 0.33, Fig. 4). Plasma amino acid concentrations are shown in Table 1. Essential amino acid concentrations, except for histidine and tryptophan, were significantly greater (2- to 10-fold greater) in the EC-AminoL40 group compared with the Sed and EC-Con groups (Table 1, *p* < 0.05). However, histidine and tryptophan concentrations were lower in the EC-AminoL40 group compared with the Sed and EC-Con groups, despite LEAA administration (Table 1, *p* < 0.05).

**Table 1:** Plasma amino acid concentrations

	Sed	EC-Con	Ec-Amino L40
Histidine	105.1 ± 5.0	110.0 ± 5.4	97.0 ± 6.5^*, +^
Isoleucine	106.8 ± 6.7	99.1 ± 6.0	409.3 ± 42.7^*, +^
Leucine	177.1 ± 10.5	160.9 ± 10.0	1652.8 ± 157.2^*, +^
Lysine	522.3 ± 33.2	474.6 ± 21.6	1240.7 ± 102.9^*, +^
Methionine	83.2 ± 5.4	82.4 ± 5.6	161.9 ± 12.0^*, +^
Phenylalanine	1337.0 ± 67.4	1327.5 ± 92.4	1368.3 ± 95.9
Threonine	487.5 ± 32.1	488.7 ± 35.1	1051.5 ± 101.7^*, +^
Tryptophan	138.4 ± 11.6	107.8 ± 6.6	101.6 ± 8.4^+^
Valine	268.4 ± 16.9	250.8 ± 14.8	891.7 ± 69.8^*, +^

Amino acid concentrations (μM) in plasma were measured 50 min following the second administration of distilled water or AminoL40. Values are mean ± SE (*n* = 9 for the Sed and EC-AminoL40 groups, and *n* = 10 for the EC-Con group)

* *p* < 0.05 significantly different from the EC-Con group

^+^ *p* < 0.05 significantly different from the Sed group

### Muscle soreness

**Fig. 5:**
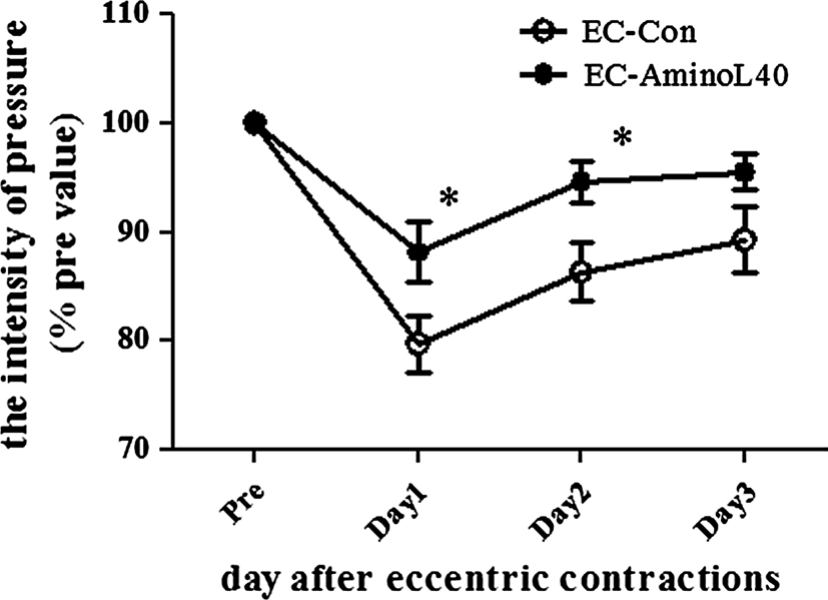
Effect of AminoL40 administration on mechanical paw withdrawal threshold after EC. The mechanical withdrawal threshold in the EC-AminoL40 group was significantly higher than in the EC-Con group on days 1 and 2. Values are shown as mean ± SE (EC-Con and EC-AminoL40 groups, *n* = 11 for each group). *p < 0.05 compared with the EC-Con group

The time course of the change in the withdrawal threshold after EC is shown in Fig. 5. The decrease in mechanical paw withdrawal threshold to a pressure stimulus was significantly suppressed in the EC-AminoL40 group at 1 and 2 days after ECs compared with the EC-Con group (*p* < 0.05, Fig. 5).

## Discussion

The objective of this study was to investigate the effect of LEAAs on the rate of muscle protein synthesis and muscle soreness after eccentric exercise in rats. First, through the comparison of the EC-Con the Sed groups (which both received distilled water) we observed that the muscle protein synthesis was blunted 60 min after EC. Second, through the comparison between EC-AminoL40 and EC-Con groups, we found that administration of LEAAs significantly alleviated the EC-induced impairment of muscle protein synthesis, and induced greater phosphorylation of p70S6K1 than EC alone. Furthermore, LEAA reduced the muscle soreness evidenced by the decrease in the mechanical paw withdrawal threshold 1 and 2 days after EC. The current results suggested that LEAA administration could alleviate the impaired muscle protein synthesis and muscle soreness induced by eccentric exercise.

We found that EC decreased muscle protein synthesis. Skeletal muscle protein synthesis is blunted 1 h after prolonged exercise in rodents (Anthony et al. 1999). However, exercise is associated with maintenance or hypertrophy of skeletal muscle and not atrophy. Therefore, the rate of muscle protein synthesis must increase during recovery from exercise. In humans, a previous study showed that skeletal muscle protein synthesis is blunted during resistance exercise. The same study also reported that an increase in AMP-activated protein kinase (AMPK) activity and a reduced phosphorylation of 4E-BP1 may contribute to the suppression of skeletal muscle protein synthesis. (Dreyer et al. 2006). It has been generally accepted that mTOR controls both 4E-BP1 and p70S6K1 directly. However, mTOR phosphorylation was unchanged immediately after exercise during the time when 4E-BP1 phosphorylation was reduced in Dreyer’s study (Dreyer et al. 2006). Furthermore, during post-exercise recovery, when muscle protein synthesis was stimulated, the increase in mTOR phosphorylation was associated with an increase in p70S6K1 phosphorylation. It suggested that the signaling mechanisms of mTOR to 4E-BP1 and p70S6K1 are regulated differentially and may be associated with the other mechanisms such as upstream regulation by TCS2 (Inoki et al. 2003). In the current study, 60 min after EC the phosphorylation of p70S6K1 was five times higher in the EC-Con than in the Sed group. However, skeletal muscle protein synthesis remained blunted. It indicated that AMPK activation or the other mechanisms which suppress the skeletal muscle protein synthesis still remained to be clear.

Notably, LEAA administration further increased the phosphorylation of p70S6K1 relatively to that induced by EC alone. Although we did not measure any other signaling molecules related to an mTOR pathway, the dose of leucine administered in our study (0.4 g/kg body weight twice, with a total dose of 0.8 g/kg body weight) has been previously reported to be sufficient to increase mTOR pathway activity (Crozier et al. 2005). Resistance exercise and amino acid administration affects muscle protein synthesis additively and BCAA administration increases the augmented phosphorylation of p70S6K1 by resistance exercise (Borgenvik et al. 2012). Therefore, different mechanisms between exercise and amino acids are proposed for stimulating the mTOR pathway. However, in Crozier’s study (Crozier et al. 2005), administration of 0.135 or 0.337 g/kg BW leucine increased the phosphorylation of p70S6K1 by four or six times its level in the control group, respectively; furthermore, skeletal muscle protein synthesis rates were not different between the groups. This suggests that, in the present study, the difference that we observed in the phosphorylation of p706SK1 between the EC-Con and EC-AminoL40 groups might not be large enough to be physiologically important. Skeletal muscle protein synthesis was higher in the EC-AminoL40 group than in the EC-Con group, but was not different from the Sed group. This indicates that the LEAA administration could partly improve the negative effect of EC on skeletal muscle protein synthesis, but the factors which suppress the muscle protein synthesis still remained. In this study, we did not include a group that received LEAA without EC because our focus was on investigating the effect of LEAAs on skeletal muscle protein synthesis rates after EC. Further studies are required to clarify the underlying discrepancy between protein synthesis and signaling.

In the current study, skeletal muscle protein synthesis and p70S6K1 phosphorylation were measured 50 min after administration of LEAAs or 60 min after ECs. Amino acid concentrations were elevated immediately, reached a peak at 30–60 min, and returned to basal levels approximately 240 min after LEAAs administration. Furthermore, Katta et al. (2009) reported that the phosphorylation of p70S6K1 was increased 0 and 1 h after ECs in rats. Thus, measurement at 50 min post-administration of LEAAs, 60 min post-ECs allowed us to detect the peak change in skeletal muscle protein synthesis and p70S6K1 phosphorylation induced by the combination of LEAA administration and ECs.

We found that LEAA administration increased plasma insulin concentrations in plasma (Fig. 3). Insulin is a powerful stimulator of protein synthesis, and leucine is known to stimulate insulin secretion. However, previous studies have reported that the stimulatory effect of leucine on muscle protein synthesis occurs without a concomitant increase in serum insulin concentrations (Anthony et al. 2000). Furthermore, a previous study demonstrated that contractile activity is a potent stimulus for blunting the net protein synthesis rate in rat skeletal muscle ex vivo and can even override the anabolic effect of insulin (Miranda et al. 2008). In our study, there was no correlation between insulin concentrations and muscle protein synthesis (Fig. 4). Therefore, an increase in insulin concentration might not have contributed to the alleviation of reduction in muscle protein synthesis seen in our study.

We found that LEAA suppressed muscle soreness after EC (Fig. 5). This is the first study to investigate the effect of amino acids on muscle soreness after EC in rats. Although muscle soreness is a typical consequence of eccentric exercise-induced muscle damage, the underlying mechanisms of muscle soreness are not clearly understood, but are probably related to the inflammatory response to muscle damage (Cheung et al. 2003). Ge et al. (2009) reported that the muscle regeneration process is regulated by the mTOR pathway. Therefore, we investigated the effect of LEAAs, as a potent mTOR stimulator, on muscle soreness. EC increased the phosphorylation of p70S6K1, and the combination of EC and LEAA administration increased the phosphorylation of p70S6K1 more than EC alone. The precise mechanisms by which LEAAs act to cause such effects remain to be determined, but LEAAs may alleviate muscle soreness, an index of muscle damage, partly via activation of the mTOR pathway. BCAAs can be transaminated to glutamate to synthesize glutamine, which is highly consumed by inflammatory cells under inflammatory conditions (Nicastro et al. 2012). Furthermore, BCAAs play a role as a precursor of glutamine and are metabolized to glutamine in skeletal muscle (Rennie and Tipton 2000). Therefore, BCAAs may affect the inflammatory status of damaged muscle by increased availability of amino acids as substrates for immune cells. Future studies should further investigate the molecular mechanisms mediated by LEAAs in the inflammatory process.

Pereira et al. reported that leucine administration improves the recovery of myofiber size and strength after cryolesion-induced muscle damage (Pereira et al. 2014). The dose of administered leucine in our study (0.4 g/kg body weight twice, for a total dose of 0.8 g/kg body weight) was less than the dose of 1.35 g/kg body weight/day used in the study conducted by Pereira et al. (2014). However, in the current study, plasma BCAA concentrations in the EC-AminoL40 group were increased by 4–10 times compared with the EC-con group (Table 1). The increase in BCAA concentrations in plasma was sufficient to activate the mTOR pathway (Crozier et al. 2005). In the current study, LEAAs stimulated the phosphorylation of p70S6K1 augmented by ECs. However, in a study conducted by Pereira et al. (2014), these beneficial effects of leucine were not associated with activation of the mTOR pathway in regenerating muscle. This difference between studies in intracellular signaling pathways remains unknown, but it could be because of the difference in the degree of muscle damage. Muscle damage was not recovered until post-cryolesion day 10 in the study conducted by Pereira et al. (2014). However, in our study, DOMS was recovered at 3 days after EC. Therefore, the degree of muscle damage might be more moderate in this exercise-induced muscle damage model than in the cryolesion model. In moderately damaged muscle, protein synthesis may be more essential for regeneration than in the greatly damaged muscle. In the current study, muscle damage and muscle function were not specifically investigated because the blood sampling and tests required to assess the muscle function could have altered the results of the pain test.

Some reports have shown that administration of amino acid, particularly BCAA, administration suppresses DOMS 1 day after exercise in humans (Jackman et al. 2010; Kirby et al. 2012; Nosaka et al. 2006). However, the mechanism by which leucine or BCAAs act to reduce the degree of muscle damage and the optimal composition to reduce the muscle soreness, remain unclear. We found that LEAAs alleviated muscle soreness after eccentric exercise in a rat model. Further studies using this animal model could help to address the questions of underlying mechanisms of leucine/BCAA effects, optimal timing of administration, and optimal composition of amino acids. In a previous study (Murase et al. 2010), the paw withdrawal threshold was decreased 1, 2, and 3 days after EC, which is similar to the change in paw withdrawal observed in this study. Furthermore, the B_2_ bradykinin receptor antagonist, HOE 140 completely suppressed the muscle soreness when injected before EC, but when injected after EC failed to reverse muscle soreness that had already developed. In the current study, the LEAAs were administered 30 min before and 10 min after EC. It remains unclear which of these administrations most affected the results. Further studies in this animal model are needed to investigate the timing of LEAA administration. We also found that LEAAs alleviated the reduction in muscle protein synthesis, which led to a positive net balance of skeletal muscle protein turnover. These results suggest that the administration of LEAAs may enhance recovery from muscle damage induced by EC. Further studies are required to determine the mechanisms responsible for the effects of BCAA or LEAA supplementation on DOMS and to investigate whether the specific composition amino acid supplementations changes these effects.

In our study, we examined LEAAs, not leucine alone or BCAAs. Administration of leucine alone or BCAAs leads to a decrease in plasma concentrations of other essential amino acids in neonatal pigs (Escobar et al. 2005) and in humans (Borgenvik et al. 2012). In normal swine, reduced availability of amino acids leads to blunted muscle protein synthesis and amino acid supplementation recovers this reduced muscle protein synthesis (Kobayashi et al. 2003). Furthermore, insulin-mediated hypoaminoacidemia reduces protein synthesis, and increased availability of all amino acids, but not leucine alone, recovers this reduction in protein synthesis. Therefore, other essential amino acids, except for BCAAs and leucine, may be essential for sustaining augmented muscle protein synthesis. Accordingly, LEAAs have been investigated to stimulate muscle protein synthesis after several types of exercise (Dreyer et al. 2008; Pasiakos et al. 2011). Additionally, in our study, we found that LEAAs alleviated muscle damage after eccentric exercise. Therefore, LEAAs are effective for recovery after exercise via alleviation of muscle damage and enhancement of muscle adaptation.

In conclusion, LEAAs suppress the decrease in mechanical paw withdrawal threshold induced by EC in rats. Additionally, LEAAs alleviate impaired muscle protein synthesis by EC and further increases phosphorylation of p70S6K1 by EC. However, the precise mechanisms by which LEAAs act to confer such effects still need to be clarified. These findings suggest that LEAA administration before and after eccentric exercise hastens the recovery from muscle soreness. Further studies are required to uncover novel mechanisms through which amino acids modulate muscle soreness and to determine whether long-term treatment with LEAAs can effectively increase muscle mass and improve muscle function.
